# Phagocytes from Mice Lacking the Sts Phosphatases Have an Enhanced Antifungal Response to Candida albicans

**DOI:** 10.1128/mBio.00782-18

**Published:** 2018-07-17

**Authors:** David Frank, Shamoon Naseem, Gian Luigi Russo, Cindy Li, Kaustubh Parashar, James B. Konopka, Nick Carpino

**Affiliations:** a Program in Molecular and Cellular Pharmacology, Stony Brook University, Stony Brook, New York, USA; b Department of Molecular Genetics and Microbiology, Stony Brook University, Stony Brook, New York, USA; c Institute of Food Sciences, National Research Council, Avellino, Italy; University of Wisconsin—Madison; Duke University Medical Center

**Keywords:** *Candida albicans*, cell signaling, effector functions, host-pathogen interactions, innate immunity

## Abstract

Mice lacking expression of the homologous phosphatases Sts-1 and Sts-2 (Sts^−/−^ mice) are resistant to disseminated candidiasis caused by the fungal pathogen Candida albicans. To better understand the immunological mechanisms underlying the enhanced resistance of Sts^−/−^ mice, we examined the kinetics of fungal clearance at early time points. In contrast to the rapid C. albicans growth seen in normal kidneys during the first 24 h postinfection, we observed a reduction in kidney fungal CFU within Sts^−/−^ mice beginning at 12 to 18 h postinfection. This corresponds to the time period when large numbers of innate leukocytes enter the renal environment to counter the infection. Because phagocytes of the innate immune system are important for host protection against pathogenic fungi, we evaluated responses of bone marrow leukocytes. Relative to wild-type cells, Sts^−/−^ marrow monocytes and bone marrow-derived dendritic cells (BMDCs) displayed a heightened ability to inhibit C. albicans growth *ex vivo*. This correlated with significantly enhanced production of reactive oxygen species (ROS) by Sts^−/−^ BMDCs downstream of Dectin-1, a C-type lectin receptor that plays a critical role in stimulating host responses to fungi. We observed no visible differences in the responses of other antifungal effector pathways, including cytokine production and inflammasome activation, despite enhanced activation of the Syk tyrosine kinase downstream of Dectin-1 in Sts^−/−^ cells. Our results highlight a novel mechanism regulating the immune response to fungal infections. Further understanding of this regulatory pathway could aid the development of therapeutic approaches to enhance protection against invasive candidiasis.

## IMPORTANCE

Systemic candidiasis caused by fungal *Candida* species is becoming an increasingly serious medical problem for which current treatment is inadequate. Recently, the Sts phosphatases were established as key regulators of the host antifungal immune response. In particular, genetic inactivation of Sts significantly enhanced survival of mice infected intravenously with Candida albicans. The Sts^−/−^ *in vivo* resistance phenotype is associated with reduced fungal burden and an absence of inflammatory lesions. To understand the underlying mechanisms, we studied phagocyte responses. Here, we demonstrate that Sts^−/−^ phagocytes have heightened responsiveness to C. albicans challenge relative to wild-type cells. Our data indicate the Sts proteins negatively regulate phagocyte activation via regulating selective elements of the Dectin-1–Syk tyrosine kinase signaling axis. These results suggest that phagocytes lacking Sts respond to fungal challenge more effectively and that this enhanced responsiveness partially underlies the profound resistance of Sts^−/−^ mice to systemic fungal challenge.

## INTRODUCTION

In recent years, an increase in the numbers of invasive infections by fungal pathogens has raised concern (1). Of particular clinical concern are diverse *Candida* species, including Candida albicans. C. albicans is responsible for a number of infectious disorders, including oral candidiasis, chronic mucocutaneous candidiasis, and invasive candidiasis, a potentially lethal infection in which the fungus disseminates systemically and proliferates within internal tissues (2). C. albicans accounts for over 50,000 hospital-acquired systemic infections in the United States alone, with a 30% to 40% mortality rate associated with the invasive form of the disease (3, 4). Current antifungal medications used to treat systemic C. albicans infections have a number of drawbacks, including high cost, toxicity, and difficulties achieving appropriate bioavailability within infected tissues (1, 5). These limitations are compounded by difficulties in making a rapid and accurate disease diagnosis (6). In addition, the emergence of drug-resistant *Candida* strains is now considered a major threat by the CDC (7, 8).

Phagocytes of the innate immune system play a critical role in the immune response to C. albicans (9). Fungal cell wall constituents are recognized by cell-surface Toll-like receptors (TLRs) and C-type lectin receptors (CLRs), promoting the activation of cellular antimicrobial effector pathways (10). However, excessive inflammatory responses that occur in the context of fungal infections can also be counterproductive and lead to detrimental collateral tissue damage (11, 12). For example, in a mouse model of systemic candidiasis, progressive sepsis caused by a vigorous inflammatory response has been identified as the cause of death (13). In this context, it has been observed that reducing inflammation by reducing levels of proinflammatory factors can lead to improved host survival (14, 15). Optimizing clinical outcomes to C. albicans infection will require a more complete understanding of the biochemical mechanisms that underlie leukocyte antifungal inflammatory responses.

We recently reported that two homologous phosphatases, Sts-1 and Sts-2, play key roles in regulating the host response to systemic C. albicans infection (16). The Sts enzymes share overlapping and redundant functions as negative regulators of hematopoietic signaling pathways (17–19). They have a distinctive structure consisting of two protein interaction domains (UBA and SH3) and a C-terminal 2H-phosphatase domain that is structurally and enzymatically very distinct from those of other intracellular protein phosphatases known to regulate immune signaling pathways (20). Within hematopoietic cell populations, Sts-1 has been shown to negatively regulate signaling downstream of the TCR by targeting the Zap-70 kinase (17) and both GPVI-FcRγ signaling in platelets and FcεRI signaling in mast cells by targeting the Zap-70 homologue Syk (21, 22).

Sts^−/−^ mice are profoundly resistant to disseminated candidiasis caused by supralethal inoculums, displaying significantly enhanced survival and an ability to clear the infection (16). To define the mechanisms underlying the enhanced resistance of Sts^−/−^ mice, we investigated the role of Sts^−/−^ leukocytes. Our results demonstrate that bone marrow-derived dendritic cells (BMDCs) lacking Sts expression have an enhanced ability to inhibit C. albicans growth and may contribute significantly to the resistance of Sts^−/−^ mice. Further, we demonstrate that, within BMDCs, the Sts phosphatases negatively regulate the activation of select pathways downstream of the key fungal pathogen pattern recognition receptor Dectin-1. These observations define a novel role for Sts in regulating host antimicrobial effector responses and provide mechanistic insights into the ability of Sts^−/−^ mice to resist lethal systemic C. albicans infection.

## RESULTS

### Inhibition of C. albicans growth within 24 h in Sts^−/−^ mice.

**FIG 1:**
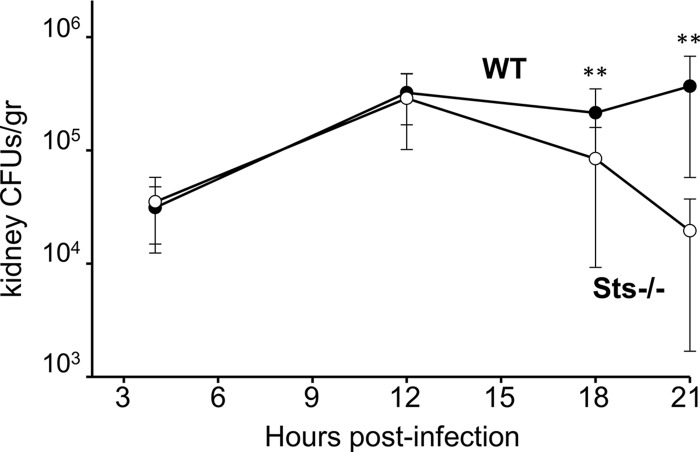
Reduced fungal CFU in Sts^−/−^ kidneys at early time points postinfection. Kidneys from mice infected with 2.5 × 10^5^ CFU were assessed for fungal burden prior to 24 h. Results represent averages of 2 to 3 independent experiments each carried out with 5 to 6 mice per group. **, *P* < 0.001 (by Mann-Whitney analysis) (error bars = standard deviations [SD] of means). WT, wild type.

In the mouse model of invasive candidiasis, the kidneys are the predominant niche for C. albicans proliferation (23). Fungal germination is evident within 2 h postinfection, and kidney fungal CFU levels increase by 2 orders of magnitude over the course of 48 h (24, 25). Previous studies showed that Sts^−/−^ mice exhibit a significantly lower kidney fungal burden that is especially evident by 48 h postinfection (16). To determine more precisely when levels of wild-type and Sts^−/−^ kidney fungal CFU begin to diverge, we compared the fungal loads in wild-type and Sts^−/−^ kidneys at early time points. Figure 1 illustrates that C. albicans within wild-type and Sts^−/−^ kidneys proliferates at similar levels in the first 12 h following infection. However, while fungal CFU levels continue to increase after 12 h in wild-type kidneys, they begin to decrease in Sts^−/−^ kidneys at between 12 and 18 h (Fig. 1). In this timeframe, similar numbers of leukocytes in wild-type and Sts^−/−^ mice have entered the kidneys (16). These data indicate that within 12 to 18 h postinfection, mice lacking the Sts proteins are more effective than wild-type mice at inhibiting fungal growth and eliminating C. albicans cells from the kidney.

### Hematopoietic stem cell (HSC)-derived cells contribute to the enhanced resistance of Sts^−/−^ mice.

**FIG 2:**
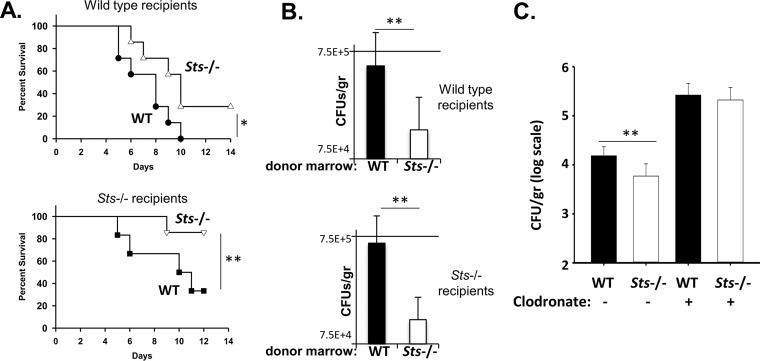
Contribution of Sts^−/−^ hematopoietic stem cell (HSC)-derived cells to the heightened resistance of Sts^−/−^ mice. (A) Radiation chimeras were infected with C. albicans (2.5 × 10^5^ CFU) by bloodstream inoculation and monitored for 28 days. Wild-type (top) or Sts^−/−^ (bottom) recipients receiving Sts^−/−^ marrow demonstrated significantly enhanced survival, as indicated. *, *P* < 0.05 (by log rank analysis). (B) Radiation chimeras were infected with C. albicans (2.5 × 10^5^ CFU) by bloodstream inoculation, and kidney CFU levels at 24 h postinfection were evaluated. Wild-type (top) or Sts^−/−^ (bottom) recipients receiving Sts^−/−^ marrow demonstrated significantly reduced 24-h kidney fungal CFU levels relative to mice receiving wild-type marrow. **, *P* < 0.01 (by Mann-Whitney analysis) (error bars = SD of means). (C) Mice treated with control liposomes (−) or a clodronate/liposome formulation (+) were infected 24 h later with 10^5^ C. albicans cells. Sts^−/−^ mice treated with clodronate failed to display enhanced fungal restriction in the kidney 24 h postinfection.

Having established that enhanced kidney fungal restriction in Sts^−/−^ animals becomes evident during the time period when bone marrow leukocyte populations begin to enter the renal compartment, we next determined if Sts^−/−^ hematopoietic cells played a role. Transplantation of Sts^−/−^ donor marrow into irradiated wild-type or Sts^−/−^ recipients enhanced survival of *Candida* infection relative to the results seen with wild-type donor cells (Fig. 2A). Additionally, irradiated wild-type and Sts^−/−^ mice reconstituted with Sts^−/−^ bone marrow displayed a significant reduction in the 24-h fungal burden relative to mice receiving wild-type bone marrow (Fig. 2B). We also noted that Sts^−/−^ recipients displayed improved survival relative to wild-type recipients given equivalent amounts of donor marrow (Fig. 2A), suggesting that a nonhematopoietic component also contributes to the increased survival of Sts^−/−^ animals. To address whether phagocytic cells play a critical role in the Sts^−/−^ resistance phenotype, we evaluated the 24-h fungal burden in mice that had been administered the phagocyte-depleting agent clodronate 24 h prior to infection (26). As expected, clodronate treatment led to higher fungal burdens. Noticeably, however, it also eliminated the 24-h fungal clearance advantage normally associated with Sts^−/−^ animals (Fig. 2C). Together, these data suggest that the hematopoietic cell compartment makes an important contribution to the enhanced resistance of Sts^−/−^ mice.

### Enhanced candidacidal activity of Sts^−/−^ leukocytes *ex vivo*.

**FIG 3:**
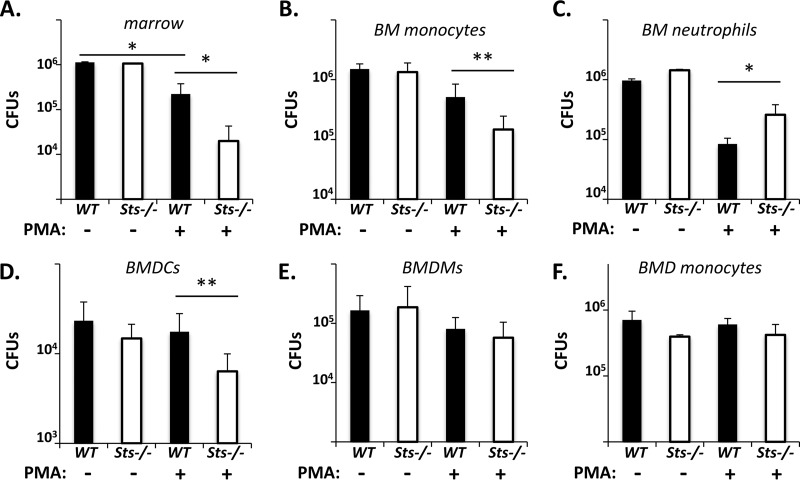
Increased antifungal activity of Sts^−/−^ phagocytes *ex vivo*. (A) Nonfilamentous C. albicans *cph1*Δ *efg1*Δ cells were coincubated with untreated or PMA-treated bone marrow cells, and fungal CFU levels were determined after 24 h. Representative results of 3 experiments performed in triplicate are displayed. (B to F) Purified (B) bone marrow monocytes, (C) bone marrow neutrophils, (D) *ex vivo*-derived BMDCs, (E) BMDMs, or (F) BMD monocytes were cultured for 24 h with fungal cells, and CFU levels were enumerated as described for panel A. Results depict the average CFU of 3 experiments performed in triplicate. **, *P* < 0.01; *, *P* < 0.05 (by Mann-Whitney analysis) (error bars = SD of means).

The regulatory role of the Sts proteins in innate leukocyte populations has not been explored. Both Sts-1 and Sts-2 are expressed by marrow cells at levels comparable to those within peripheral blood leukocytes and splenic cells (see Fig. S1A in the supplemental material). Therefore, we utilized an *in vitro* cell/fungal coculture assay to examine directly the interaction of bone marrow cells with fungal cells *ex vivo* (27). Marrow cells isolated from uninfected wild-type and Sts^−/−^ mice were placed in culture and incubated with C. albicans *cph1Δ efg1Δ* cells. The *cph1Δ efg1Δ* mutant was used because it fails to undergo hyphal growth (28), thereby facilitating accurate quantification of fungal growth. After 24 h, we recovered equivalent CFU levels in cocultures containing untreated wild-type or Sts^−/−^ marrow cells (Fig. 3A). In the presence of the immune cell activator phorbol myristate acetate (PMA), fewer fungal CFU were obtained (Fig. 3A). Significantly, PMA-treated Sts^−/−^ marrow cells were more efficient at inhibiting fungal growth than treated wild-type marrow cells (Fig. 3A). This suggests that the antifungal properties of PMA-treated bone marrow cells are potentiated in the absence of Sts expression.

10.1128/mBio.00782-18.1 FIG S1  Expression of Sts-1 and Sts-2 in murine hematopoietic cells. Representative Western blots illustrate levels of Sts-1 and Sts-2 in (A) bone marrow (BM), blood, and spleen; (B) purified monocytes (Mo) isolated from preparations of marrow cells; (C) purified neutrophils (Neu) isolated from heterogeneous marrow populations; (D) BMDMs derived by culturing marrow cells in CSF-1 over a 5-day period and removing nonadherent cells; (E) BMD monocytes derived by culturing marrow cells in CSF-1 over a 4-day period and harvesting nonadherent cells; and (F) BM-derived dendritic cells (BMDCs) derived by culturing cells in GM-CSF for 10 days, harvesting nonadherent cells, and purifying them by flow cytometry (CD45^+^ CD11b^+^ major histocompatibility complex class II^+^ [MHCII^+^] CD11c^+^). Download FIG S1, TIF file, 1.9 MB. Copyright © 2018 Frank et al. 2018 Frank et al. This content is distributed under the terms of the Creative Commons Attribution 4.0 International license.

Marrow cell types associated with the initial innate immune response to systemic C. albicans infection include neutrophils and monocytes (29–31). Sts-1 and Sts-2 were found to be highly expressed in both cell populations (Fig. S1B and C [for cell purity data, see Fig. S2]). In order to examine the role of Sts in the antifungal responses of bone marrow monocytes and neutrophils, cells were purified from preparations of murine marrow. Sts^−/−^ marrow monocytes exhibited 10-fold-greater inhibition of fungal growth than the corresponding wild-type cultures (Fig. 3B). As in the case of total bone marrow cell coculture, the increased fungal-growth-inhibitory properties displayed by Sts^−/−^ monocytes was evident only when cells were pretreated with PMA. In contrast to marrow monocytes, PMA-treated neutrophils lacking Sts expression were less efficient at inhibiting fungal growth than wild-type neutrophils, although both were better at inhibiting fungal growth than untreated cells (Fig. 3C). We also examined the different phagocyte populations that can be obtained by culturing bone marrow *ex vivo* in the presence of different cytokines. These include bone marrow-derived dendritic cells (BMDCs) (32), bone marrow-derived macrophages (BMDMs) (33), and bone marrow-derived monocytes (BMD monocytes) (34) (see Fig. S1D to F). Similarly to bone marrow cells and marrow monocytes, Sts^−/−^ BMDCs exhibited a significantly greater ability to restrict C. albicans growth *ex vivo* than wild-type BMDCs (Fig. 3D). In contrast, no differences were observed in the growth-inhibitory properties of wild-type and Sts^−/−^ BMDMs and BMD monocytes (Fig. 3E and F). Cumulatively, our results suggest that the Sts proteins play a negative role in regulating antifungal properties of select phagocyte populations.

10.1128/mBio.00782-18.2 FIG S2  Isolation of hematopoietic bone marrow cells. Representative fluorescence-activated cell sorter (FACS) plots of the (A) purified Ly6C^hi^ bone marrow monocytes or (B) Ly6G^+^ bone marrow neutrophils used for *ex vivo* coculture assays (Fig. 2) are shown. Download FIG S2, TIF file, 0.8 MB. Copyright © 2018 Frank et al. 2018 Frank et al. This content is distributed under the terms of the Creative Commons Attribution 4.0 International license.

### Increased *Candida*-induced ROS production in cells lacking Sts expression.

**FIG 4:**
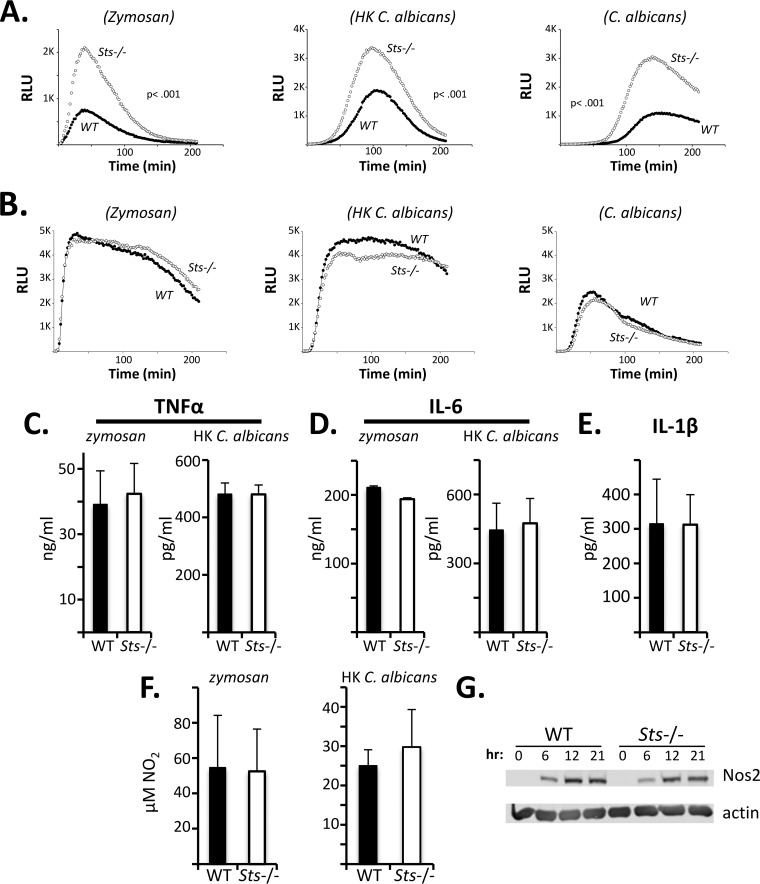
Increased *Candida*-induced ROS production in Sts^−/−^ BMDCs. (A) Wild-type and Sts^−/−^ BMDCs were stimulated with zymosan, heat-killed (HK) C. albicans, or live C. albicans as indicated, and levels of ROS production were assessed by luminol chemiluminescence. Average results of at least three separate experiments each conducted in triplicate are displayed. *P* values for the areas under each curve were calculated by Mann-Whitney analysis. RLU, relative light units. (B) Wild-type and Sts^−/−^ neutrophils were stimulated as described for panel A and levels of ROS production assessed. (C) Equivalent levels of TNF-α in wild-type and Sts^−/−^ BMDC culture supernatants stimulated with zymosan or HK C. albicans for 24 h, as indicated. Combined results of at least three separate experiments each conducted in duplicate are displayed. (D) Equivalent levels of IL-6 in wild-type and Sts^−/−^ BMDC culture supernatants stimulated with zymosan or HK C. albicans for 24 h, as indicated. Representative (zymosan) or combined (HK C. albicans) results of at least three separate experiments each conducted in duplicate are displayed. (E) Equivalent levels of IL-1β in wild-type and Sts^−/−^ BMDC culture supernatants stimulated for 24 h with 100 µg/ml zymosan. (F) Equivalent levels of nitrite, representative of NO production, in wild-type and Sts^−/−^ BMDC culture supernatants stimulated with zymosan (left) or HK C. albicans (right) for 24 h. Combined results of at least three separate experiments each conducted in duplicate are displayed. (G) Induction of Nos2 in zymosan-stimulated BMDCs. Representative results of two separate experiments are displayed. (Error bars for panels C to F = SD of means).

Because Sts^−/−^ BMDCs displayed enhanced fungal growth suppression *ex vivo* (Fig. 3D), we next investigated their antifungal effector functions. Members of the C-type lectin receptor (CLR) superfamily, including Dectin-1, are among the surface receptors engaged by fungal cells (35, 36). We stimulated cells with zymosan, a crude preparation of yeast cell wall extract that engages antifungal TLRs and CLRs, and observed a significantly greater zymosan-induced reactive oxygen species (ROS) response in Sts^−/−^ BMDC cultures than in wild-type BMDCs. In particular, both the rate of ROS production and the peak ROS signal were significantly enhanced in cells lacking Sts (Fig. 4A, left). We then evaluated the ROS response of BMDCs treated with C. albicans cells. After addition of either live or heat-killed (HK) C. albicans to BMDC cultures, production of ROS became evident, although the onset of the ROS response was delayed relative to cells treated with zymosan (Fig. 4A, middle and right panels). Similarly to the response seen following zymosan treatment, ROS production by Sts^−/−^ BMDCs following challenge with fungal cells was significantly augmented relative to the ROS response of wild-type BMDCs. In contrast to BMDCs, wild-type and Sts^−/−^ neutrophils (Fig. 4B) and BMD monocytes (Fig. S3A) did not display any differences in fungus-induced ROS production, while BMDMs of both genotypes did not generate a fungus-induced ROS response (Fig. S3B). Stimulation of BMDCs with heat-killed C. albicans *cph1Δ efg1Δ* cells also produced a heightened ROS response in Sts^−/−^ cells relative to wild-type cells (Fig. S4). Together, our data indicate that the Sts proteins negatively regulate the activation of fungus-induced ROS production in BMDCs.

10.1128/mBio.00782-18.3 FIG S3  Sts does not regulate ROS production in BMD monocytes or BMDMs. Wild-type and Sts^−/−^ (A) marrow-derived monocytes and (B) BMDMs were stimulated with zymosan, HK C. albicans, or live C. albicans as indicated, and levels of ROS production were assessed by luminol chemiluminescence. Average results of at least two separate experiments each conducted in triplicate are displayed. Download FIG S3, TIF file, 1.5 MB. Copyright © 2018 Frank et al. 2018 Frank et al. This content is distributed under the terms of the Creative Commons Attribution 4.0 International license.

10.1128/mBio.00782-18.4 FIG S4  Increased ROS production from heat-killed nonfilamentous C. albicans. (Left) Wild-type and Sts^−/−^ BMDCs were stimulated with HK C. albicans *cph1*Δ *efg1*Δ (MOI of 50), and levels of ROS production were assessed by luminol chemiluminescence. (Right) Average results (areas under the curve [AUC]) of two separate experiments each conducted in triplicate are displayed. **, *P* < 0.01 (by Mann-Whitney analysis) (error bars = SD of means). Download FIG S4, TIF file, 0.4 MB. Copyright © 2018 Frank et al. 2018 Frank et al. This content is distributed under the terms of the Creative Commons Attribution 4.0 International license.

In addition to ROS production, fungus-stimulated phagocytes produce diverse proinflammatory cytokines, including tumor necrosis factor alpha (TNF-α), interleukin-6 (IL-6), and IL-1β (37). In the case of the latter, activation of the NLRP3 inflammasome downstream of the fungal receptor Dectin-1 results in upregulation of IL-1β secretion (38). In contrast to the differential ROS responses observed between wild-type and Sts^−/−^ BMDCs, we detected no differences in the production of TNF-α, IL-6, or IL-1β by cells lacking Sts expression relative to wild-type cells (Fig. 4C to E). Additionally, wild-type and Sts^−/−^ cells upregulated Nos2 expression to similar extents following stimulation, resulting in identical levels of NO production (Fig. 4F and G). These results suggest that the Sts proteins regulate a subset of phagocyte antifungal effector functions that includes the production of reactive oxygen species.

### Sts regulates ROS production downstream of fungal receptor Dectin-1.

**FIG 5:**
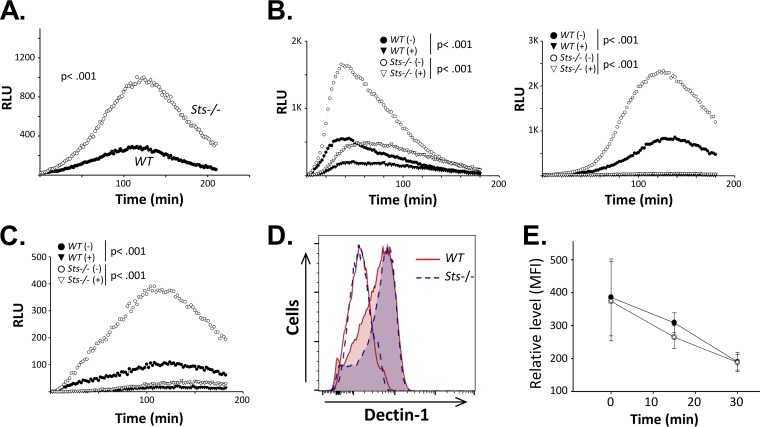
Sts regulates ROS production downstream of fungal CLR Dectin-1. (A) Wild-type and Sts^−/−^ BMDCs were stimulated with particulate β-glucan, and then levels of ROS production were assessed by luminol chemiluminescence. (B) Zymosan-induced (left) or HK (heat-killed) wild-type C. albicans-induced (right) ROS production in wild-type and Sts^−/−^ BMDCs was assessed in the absence (−) or presence (+) of soluble β-glucan, a specific inhibitor that blocks activation of Dectin-1 signaling pathways (46). (C) ROS production in wild-type and Sts^−/−^ BMDCs was assessed in the absence (−) or presence (+) of a blocking anti-Dectin-1 antibody. (D) Equivalent levels of surface Dectin-1 receptor expressed on wild-type BMDCs (solid red line) and Sts^−/−^ BMDCs (dotted dark blue line), evaluated by flow cytometry with a specific anti-Dectin-1 antibody. Nonshaded lines represent a nonspecific rat IgG control. (E) Equivalent levels of zymosan stimulation-dependent downregulation of surface Dectin-1 on wild-type (closed dot) and Sts^−/−^ (open dot) BMDCs. MFI, mean fluorescence intensity. For panels A to C, average values from at least 3 independent experiments each performed in triplicate are presented. *P* values were calculated by Mann-Whitney analysis.

Dectin-1 is a CLR that is stimulated by C. albicans and mediates activation of numerous downstream pathways (39, 40). Among the ligands that stimulate Dectin-1 are polymeric forms of β-glucan that are components of fungal cell walls (10). To investigate the involvement of Dectin-1, we treated BMDCs with purified particulate β-1,3 glucan polymers and found that Sts^−/−^ BMDCs demonstrated significantly increased ROS production relative to wild-type cells (Fig. 5A). Unlike polymeric β-glucan, soluble monomeric β-glucan acts in a competitive inhibitory fashion and blocks access to the Dectin-1 ligand-binding surface (41). After addition of soluble β-glucan to BMDC cocultures, the enhanced ROS response of Sts^−/−^ cells following zymosan or HK C. albicans stimulation was inhibited (Fig. 5B). The elevated ROS response of Sts^−/−^ BMDCs was also abrogated following addition of a blocking anti-Dectin-1 antibody to cocultures but not following addition of a control antibody (Fig. 5C). Importantly, we noted no difference in levels of surface expression of Dectin-1 on wild-type and Sts^−/−^ BMDCs (Fig. 5D). Similarly, no differences in the stimulation-dependent downregulation of Dectin-1 from the cell surface were observed (Fig. 5E). These results suggest that the Sts proteins act downstream of Dectin-1 to negatively regulate activation of the fungus-induced ROS response.

### Sts regulates levels of Syk activation induced by C. albicans.

**FIG 6:**
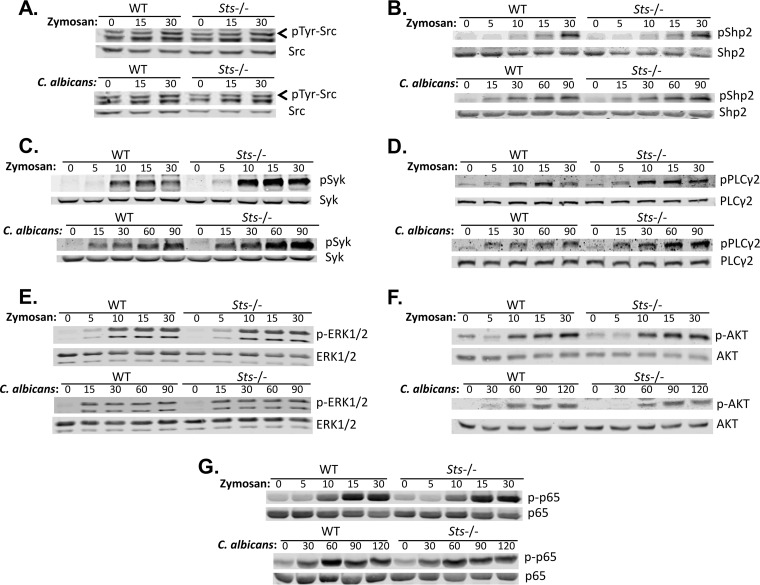
Increased activation of Syk and PLCγ2 downstream of Dectin-1. Wild-type or Sts^−/−^ BMDCs were stimulated with either zymosan or live C. albicans (MOI of 0.5) for the indicated times, and levels of activation of (A) Src kinases; (B) Shp2 phosphatase; (C) Syk; (D) PLCγ2; (E) ERK1/2; (F) Akt; and (G) p65 NF-κB were determined with phosphospecific antibodies. Representative results from 3 independent experiments for each stimulation are displayed. Results for Syk and PLCγ2 were quantified and analyzed for statistical significance (see Fig. S5).

Among the first known biochemical events following Dectin-1 stimulation are upregulation of Src family member kinase activity and phosphorylation of Shp2 phosphatase (42, 43). Spleen tyrosine kinase (Syk), a nonreceptor protein kinase that is highly expressed in a variety of phagocytes, is then activated (44). We examined activation levels of Src family kinases in stimulated wild-type and Sts^−/−^ cells using a phosphospecific antibody. No differences were observed in the levels of Src kinase activation following stimulation of BMDCs with zymosan or wild-type C. albicans*.* (Fig. 6A). Further, no differences between stimulated wild-type and Sts^−/−^ cells in Shp2 phosphorylation levels were noted (Fig. 6B). In contrast, stimulation of wild-type and Sts^−/−^ cells with either zymosan or C. albicans resulted in enhanced Syk tyrosine phosphorylation in Sts^−/−^ cells (Fig. 6C; quantified in Fig. S5A). These observations suggest that the Sts phosphatases regulate Dectin-1 signaling at the level of Syk phosphorylation.

10.1128/mBio.00782-18.5 FIG S5  Increased phosphorylation of signaling molecules downstream of Dectin-1. Levels of site-specific phosphorylation at (A) Syk Tyr-525/526 and (B) PLCγ2 Tyr-759 in zymosan-stimulated (top) or wild-type C. albicans-stimulated (bottom) BMDCs were determined by quantifying the fluorescent intensities of protein bands using a Li-COR Odyssey imaging system and normalizing to the level of total Syk or PLCγ2 protein, respectively. Each measurement was expressed as a value relative to the normalized level of site-specific phosphorylation observed in wild-type cells. The illustrated data represent averages of results from three to four separate experiments. **, *P* < 0.01; *, *P* < 0.05 (by Student’s *t* test). Download FIG S5, TIF file, 0.9 MB. Copyright © 2018 Frank et al. 2018 Frank et al. This content is distributed under the terms of the Creative Commons Attribution 4.0 International license.

Engagement of Dectin-1 by fungus-associated ligands leads to activation of a number of intracellular signaling pathways. To investigate pathways downstream of Syk in Sts^−/−^ phagocytes, we examined activation of PLCγ2, a downstream substrate of Syk (40). PLCγ2 was found to be hyperphosphorylated in stimulated Sts^−/−^ BMDCs relative to wild-type cells (Fig. 6D; quantified in Fig. S5B). We also examined the activation of extracellular signal-regulated kinase-1/2 (ERK1/2) and phosphatidylinositol 3-kinase (PI3K), signaling molecules that play critical roles in transcriptional activation downstream of C-type lectin receptors. No differences were observed in the kinetics or extent of ERK1/2 activation (Fig. 6E), or in PI3K activation, as indicated by the kinetics and extent of Akt phosphorylation (Fig. 6F). Finally, levels of activation of p65 NF-κB in wild-type and Sts^−/−^ cells following stimulation with either zymosan or C. albicans were identical (Fig. 6G). These results suggest that the Sts proteins could regulate a specific Syk-PLCγ2-ROS signaling axis downstream of Dectin-1 that is independent of the pathways regulating Dectin-1/Syk-induced cytokine gene expression.

## DISCUSSION

Combination therapy that pairs the use of traditional antibiotics with agents to enhance beneficial host immune responses is considered an important therapeutic goal for the treatment of intractable infections and of those for which antibiotic resistance is a looming concern (11, 12). Interestingly, genetic inactivation of the Sts proteins dramatically improves host survival following lethal doses of intravenous C. albicans, suggesting that they are possible targets to enhance host antifungal immunity. Importantly, the resistance of Sts^−/−^ mice is accompanied by rapid fungal clearance within the kidney, sharply decreased levels of inflammatory molecules, and an absence of inflammatory lesions. While our previous analysis revealed key differences in the immunological responses of wild-type versus Sts^−/−^ mice (16), it did not provide mechanistic insights into how Sts inactivation alters immune responses and increases protection from systemic infection. In this study, therefore, we sought to identify underlying cellular and molecular components of the enhanced antifungal immune response displayed by Sts^−/−^ mice.

### Increased resistance to systemic C. albicans is mediated by Sts^−/−^ leukocytes.

Comparative analyses of fungal clearance within infected mice provided an important clue into underlying mechanisms. Critically, C. albicans grew similarly in wild-type and Sts^−/−^ kidneys during the first 12 h postinfection. As kidney-resident phagocytes represent the primary innate immune cell population within uninfected kidneys (45), this observation suggests that the initial responses of resident phagocytes do not account for the differential abilities of wild-type and Sts^−/−^ mice to control the infection. However, levels of fungal CFU within infected kidneys begin to differ between 12 and 18 h postinfection (Fig. 1), with Sts^−/−^ CFU beginning to decline at a time when large numbers of leukocytes enter the renal compartment to counteract the infection (23, 31). This suggests that a key contribution is made by Sts^−/−^ bone marrow-derived leukocytes, a hypothesis that is supported by our radiation chimera studies in which animals reconstituted with Sts^−/−^ marrow had improved fungal clearance and enhanced survival relative to animals receiving wild-type marrow (Fig. 2). *Ex vivo* coculture analysis also demonstrated increased fungal growth suppression associated with Sts^−/−^ phagocytes (Fig. 3). Together, these observations suggest that the Sts phosphatases negatively regulate intrinsic antifungal responses within key leukocyte populations.

### Increased ROS production downstream of Dectin-1–Syk signaling in Sts^−/−^ cells.

After stimulating BMDCs with fungal ligands, we observed significantly heightened ROS production in Sts^−/−^ cells but no differences in other antifungal responses such as cytokine production or generation of nitric oxide (Fig. 4). ROS production is known to be induced following engagement of the CLR Dectin-1 (46), and we confirmed the involvement of Dectin-1 using both a competitive inhibitor and blocking antibodies. While the signaling pathway from Dectin-1 to initiation of the ROS response has not been fully elucidated, one established component is the Syk kinase (38, 44, 47). Using a phosphospecific antibody that recognizes the tyrosine phosphorylated activation loop of Syk, we observed hyperphosphorylation of Syk in Sts^−/−^ BMDCs following stimulation with zymosan or infection with C. albicans (Fig. 6). Under these conditions, there was no evidence of increased activation of upstream components such as Src kinases or Shp2. Therefore, our data suggest that Sts regulates signaling events downstream of Dectin-1 at the level of Syk phosphorylation and activation, perhaps by direct dephosphorylation of Syk. The idea of a role for Sts in regulating Syk activity in BMDCs is supported by studies in other cell types that demonstrated that Syk is a Sts target (19, 21, 22).

Further evidence for heightened Syk kinase activity in Sts^−/−^ BMDCs lies in the observation that PLCγ2, a putative Syk substrate, displays increased phosphorylation following stimulation of the Dectin-1 pathway. Interestingly, we did not observe any differences in the levels of activation of the mitogen-activated protein kinase (MAPK) and PI3K pathways, two signaling pathways also thought to be downstream of Syk (48). We also observed no differences between wild-type and Sts^−/−^ cells in production of TNF-α, IL-6, and IL-1β, which are three cytokines that have been shown to lie downstream of the location of Syk activation (47). Together, these data highlight complexity in the regulation of Syk signaling that heretofore has not been described. In particular, the distinct effects on downstream effector pathways in Sts^−/−^ cells suggest that Syk-mediated activation of downstream pathway components occurs in a differentially regulated manner. Whether this involves interaction of activated Syk with multiple distinct regulatory factors or differential subcellular localization of activated Syk is currently unclear. How Sts deficiency and increased Syk activation together lead selectively to increased ROS production is currently being investigated.

### Cell-specific regulation of antifungal responses by Sts.

Interestingly, our *ex vivo* analysis data suggest that Sts regulates leukocytes in a cell-specific manner. In particular, while Sts^−/−^ BMDCs displayed increased candidacidal activity, BM-derived macrophages and BM-derived monocytes were unaffected by Sts inactivation. Furthermore, although bone marrow monocytes and neutrophils both expressed high levels of the Sts proteins, only Sts^−/−^ monocytes displayed enhanced C. albicans growth-suppressive properties. The underlying basis for a cell-specific role for Sts in regulating antifungal responses is currently unclear, but it could indicate important differences in the manner in which different innate immune cells respond to fungal pathogens. These observations are consistent with a previous report from a study demonstrating that different bone marrow-derived cell lineages exhibit differential responses to fungal ligands (49). It will be interesting to determine how the cell specificity observed *ex vivo* influences the *in vivo* immune response to fungal infection.

### Increasing resistance to C. albicans infection.

Similarly to the Sts proteins, two other gene products (Jnk1 and Cbl-b) have recently been shown to negatively regulate phagocyte signaling pathways such that the corresponding gene deletions result in mice that have increased resistance to C. albicans bloodstream infection (12, 50–53). Jnk1, a member of the MAPK family of enzymes, negatively regulates activation of the transcription factor NFATc1. NFATc1 induces expression of the CLR CD23, and CD23 expression is upregulated in Jnk1^−/−^ mice phagocytes, resulting in elevated levels of inducible nitric oxide synthase (iNOS) (Nos2) expression. Therefore, the protection of Jnk1^−/−^ mice from systemic candidiasis appears to stem from increased fungus-induced NO production (50). The ubiquitin ligase Cbl-b mediates Dectin-1 internalization and degradation. In its absence, Dectin-1 surface expression is stabilized, resulting in Syk hyperactivation and enhanced phagocyte antifungal responses (51–53). Interestingly, while Cbl-b^−/−^ BMDCs demonstrate increases in both ROS and cytokine production levels following infection with C. albicans, Sts^−/−^ cells display an augmented ROS response without concomitant increases in cytokine production. Nonetheless, an intriguing property common to Jnk1^−/−^, Cbl-b^−/−^, and Sts^−/−^ mutant mice is increased activation of phagocyte signaling pathways downstream of fungal CLRs, with consequent increases in antifungal effector activities. These observations offer insights into developing novel immune-enhancing therapeutics that could be paired with traditional antifungal antibiotics to ameliorate the destructive effects of systemic C. albicans infection.

## MATERIALS AND METHODS

### Mouse strains and cells.

The generation of C57/B6 mice containing the Sts mutations has been previously described (17, 54, 55). Mice were housed in the Stony Brook University Animal Facility in accordance with Division of Laboratory Animal Resources (DLAR) regulations. Animal protocols followed guidelines established within the “Guide for the Care and Use of Laboratory Animals" (8th ed.) published by the National Research Council of the National Academies. Protocols were approved by the Institutional Animal Care and Use Committee (IACUC) of Stony Brook University.

BMDCs were differentiated as previously described (32). Briefly, cells were cultured in RPMI medium containing 10% fetal bovine serum (FBS), 1 mM sodium pyruvate, 10 U/ml penicillin/streptomycin (Pen/Strep), 55 µM β-mercaptoethanol (BME), and 20 ng/ml granulocyte-macrophage colony-stimulating factor (GM-CSF). On days 3, 6, and 8, cells were provided fresh growth media. All BMDC experiments utilized nonadherent cells grown for 9 to 10 days in culture. BMDMs were cultured in DMEM containing 30% L929 cell supernatant (33), 20% FBS, and 1 mM sodium pyruvate for 4 days, after which cells were harvested, counted, and utilized as described. During the derivation of BMDMs, day 4 nonadherent cells were harvested as BM-derived monocytes (34).

For neutrophil purification, bone marrow cells were suspended in 4 ml of phosphate-buffered saline (PBS), placed on 3 ml Lymphoprep reagent (Axis-Shield, Oslo, Norway) (1.077 g/ml), and spun at 2,000 rpm. Alternatively, a murine neutrophil enrichment kit (Miltenyi) was utilized to obtain marrow neutrophils. Bone marrow monocytes were obtained with a murine monocyte isolation kit (Miltenyi) or an EasySep mouse monocyte kit (Stem Cell Technologies), according to the instructions of the manufacturers.

### Reagents and antibodies.

The following antibodies were purchased from Cell Signaling Technology, Inc.: pAKT S473 (9271), AKT (9272), p-ERK T202/Y204 (9106), ERK (9102), p-Syk Y525/526 (2710), p-PLCγ2 Y759 (3874), p-Src family Y416 (2101), Src (2110), p-Shp2 Y542 (3715), Shp2 (3397), p-P65 S536 (3033), and P65 (6956). Anti-Syk (Syk01) and anti-Nos2 (5C1B52) antibodies were from BioLegend. Antibodies to PLCγ2 (sc5283) were from Santa Cruz Biotechnology. Antibodies to Sts-1 and Sts-2 were previously described (17, 54). Dectin-1 antibody (2A11) was from Bio-Rad, and flow cytometry antibodies to CD45 (clone 30-F11), CD11b (clone M1/70), CD11C (clone N418), F4/80 (clone BM8), Ly6g (clone 1A8), Ly6c (clone AL-21), and I-A/I-E (clone M5/114.15.2) were from BioLegend. Luminol, PMA, horseradish peroxidase (HRP) type VI (P8375), and zymosan (Z4250) were from Sigma-Aldrich. Particulate β-glucan, soluble β-glucan, and depleted zymosan were obtained from InvivoGen.

### Mouse infections.

Candida albicans infections were carried out as previously described (16). Cells were harvested, washed twice in PBS, and counted, and cell counts were confirmed by plating dilutions onto yeast extract-peptone-dextrose (YPD) plates. Female mice were inoculated with 2.5 × 10^5^ CFU via the lateral tail vein and monitored for 28 days. For clodronate depletion experiments, mice were administered by intravenous (i.v.) injection 1 mg clodronate/liposome formulation or control liposomes (Encapsula Nano Sciences, Brentwood, TN) 24 h prior to inoculation with C. albicans (1 × 10^5^ CFU) (26). To obtain kidney CFU, kidneys were excised at the indicated times postinfection, placed in 5 ml PBS, and homogenized. The number of CFU per gram of tissue was determined by plating homogenate serial dilutions onto YPD medium plates and incubating at 30°C.

### Derivation and use of radiation chimeras.

Female mice (8 to 10 weeks of age) were dosed with 1,100 rads from a gamma cell irradiator (GammaCell 40; AEC Ltd.) and administered 8 × 10^6^ bone marrow cells via tail vein injection within 1 h. Chimeric mice were housed for 12 weeks and then utilized for survival or organ CFU assays. Graphing and statistical analysis of survival after infection were carried out using a log rank test (Mantel-Haenszel test) with SigmaPlot software (SigmaPlot Systat Software, Inc., San Jose, CA).

### *Ex vivo* C. albicans coculture assay.

Nonfilamentous C. albicans mutant *cph1*Δ *efg1*Δ cells (28) were grown overnight, reinoculated into fresh medium, and grown to an optical density at 600 nm (OD_660_) of 0.7 to 0.9. Cells were washed twice in PBS and coincubated with cells obtained from male or female mice in RPMI media at a multiplicity of infection (MOI) of 0.0375 to 0.125, with or without 10 ng/ml PMA, for 24 h in a 96-well plate. Wells were washed once with water and collected in 1 ml of deionized water to lyse nonfungal cells. Fungal CFU were obtained by plating serial dilutions onto YPD plates.

### Pathway analysis.

Cells obtained from male or female mice were stimulated, washed, and lysed in buffer containing 0.05 M Tris, 0.15 M sodium chloride, 5 µM EDTA, 0.2 mM pervanadate, 0.5 mg/ml phenylmethylsulfonyl fluoride (PMSF), and 1× Roche protease inhibitors. Lysates were clarified by centrifugation, subjected to SDS-PAGE, and transferred to nitrocellulose (Whatman). Membranes were probed with specific antibody and the appropriate secondary antibody and were developed with an Odyssey CLx imaging system (Li-COR). Immunoprecipitations were conducted by rotating lysates with specific antibody for 2 h at 4°C, followed by 1 h at 4°C with protein A Sepharose beads (Sigma). Beads were washed three times in lysis buffer, and proteins were eluted with 2× Laemmli sample buffer and separated by SDS-PAGE. Dectin-1 downregulation was evaluated using a FACScan cytometer (Cytek Biosciences).

### Measurement of ROS and NO production.

Levels of reactive oxygen species were measured as previously described (56). Briefly, BMDCs (1 × 10^5^ cells/well) or neutrophils (4 × 10^5^ cells/well) were plated in triplicate wells of a 96-well plate. Stimuli were prepared in RPMI media containing 600 µM luminol and 16 units of HRP/ml. Reagent medium (100 μl) was added to 100 µl of preplated cells, and luminescence was measured at regular intervals on a Filtermax F5 96-well plate reader using Softmax Pro software (Molecular Devices, Sunnyvale, CA). Griess reagent was used to evaluate levels of NO_2_ production in cell culture supernatants per the instructions of the manufacturer (Promega).

### Cytokine measurements.

Cells were placed in a 6-well tissue culture plate and stimulated with zymosan (100 µg/ml) or heat-killed SC5314 (MOI of 2). The supernatant was collected and frozen at −80°C until measured. IL-6, TNF-α (BioLegend), and IL-1β (Thermo Fisher Invitrogen) levels were measured by enzyme-linked immunosorbent assay (ELISA) according to provided instructions.
