# Supplementary figures and images for: Phagocytes from Mice Lacking the Sts Phosphatases Have an Enhanced Antifungal Response to Candida albicans
Source: mBio. 2018 Jul 17;9(4):e00782-18. doi: 10.1128/mBio.00782-18 (PMC6050958; doi:10.1128/mBio.00782-18)

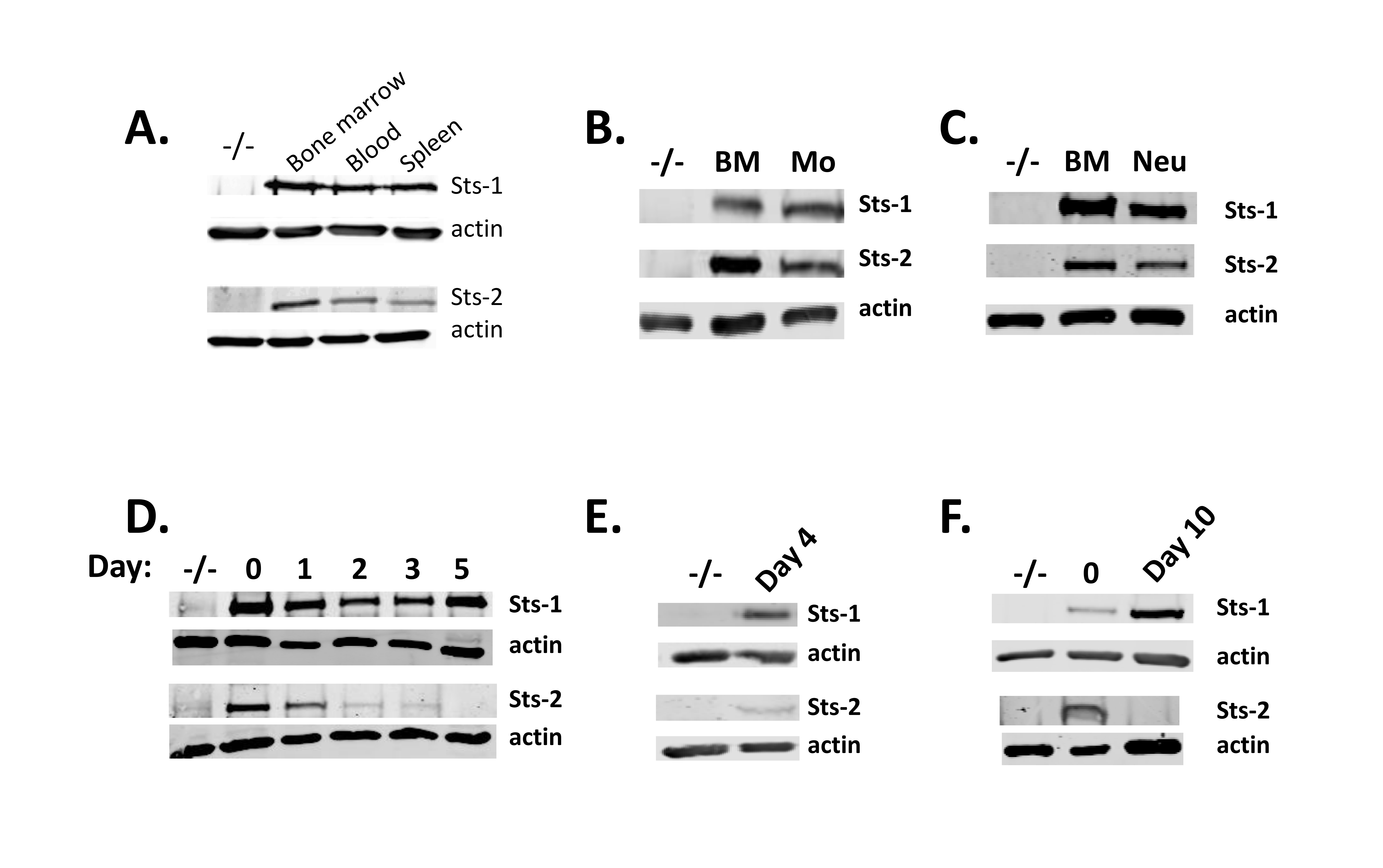

Supplement: FIG S1 [file mbo004183975sf1.tif]

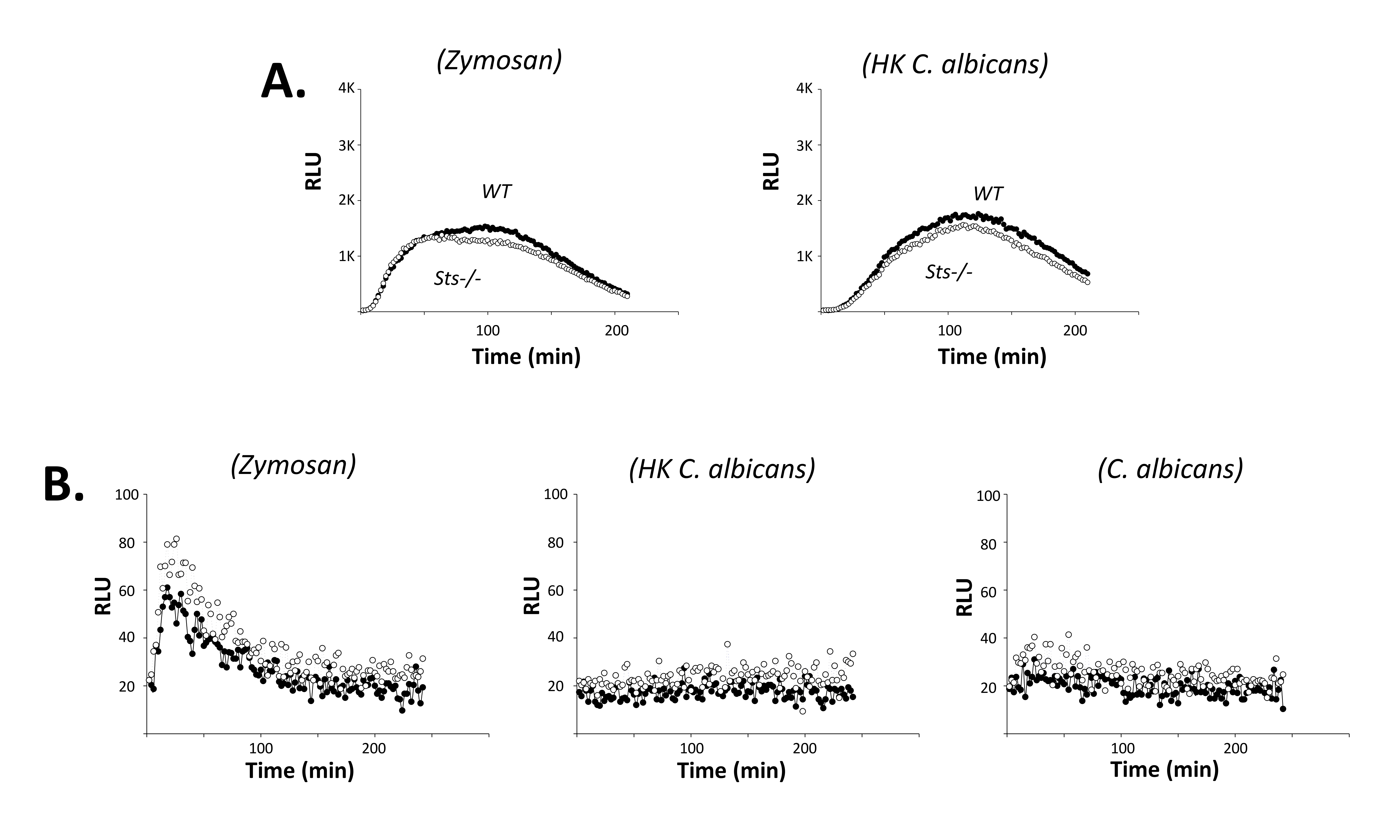

Supplement: FIG S3 [file mbo004183975sf3.tif]

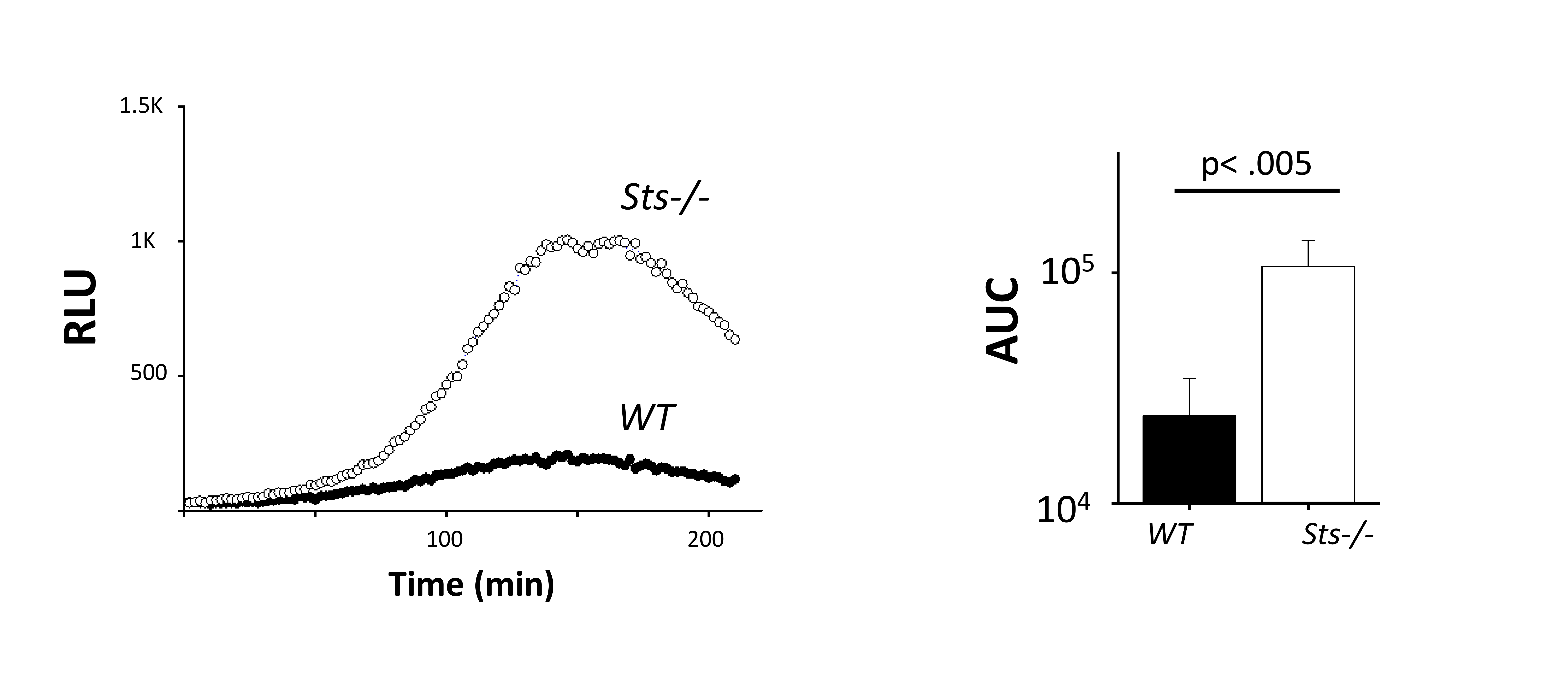

Supplement: FIG S4 [file mbo004183975sf4.tif]

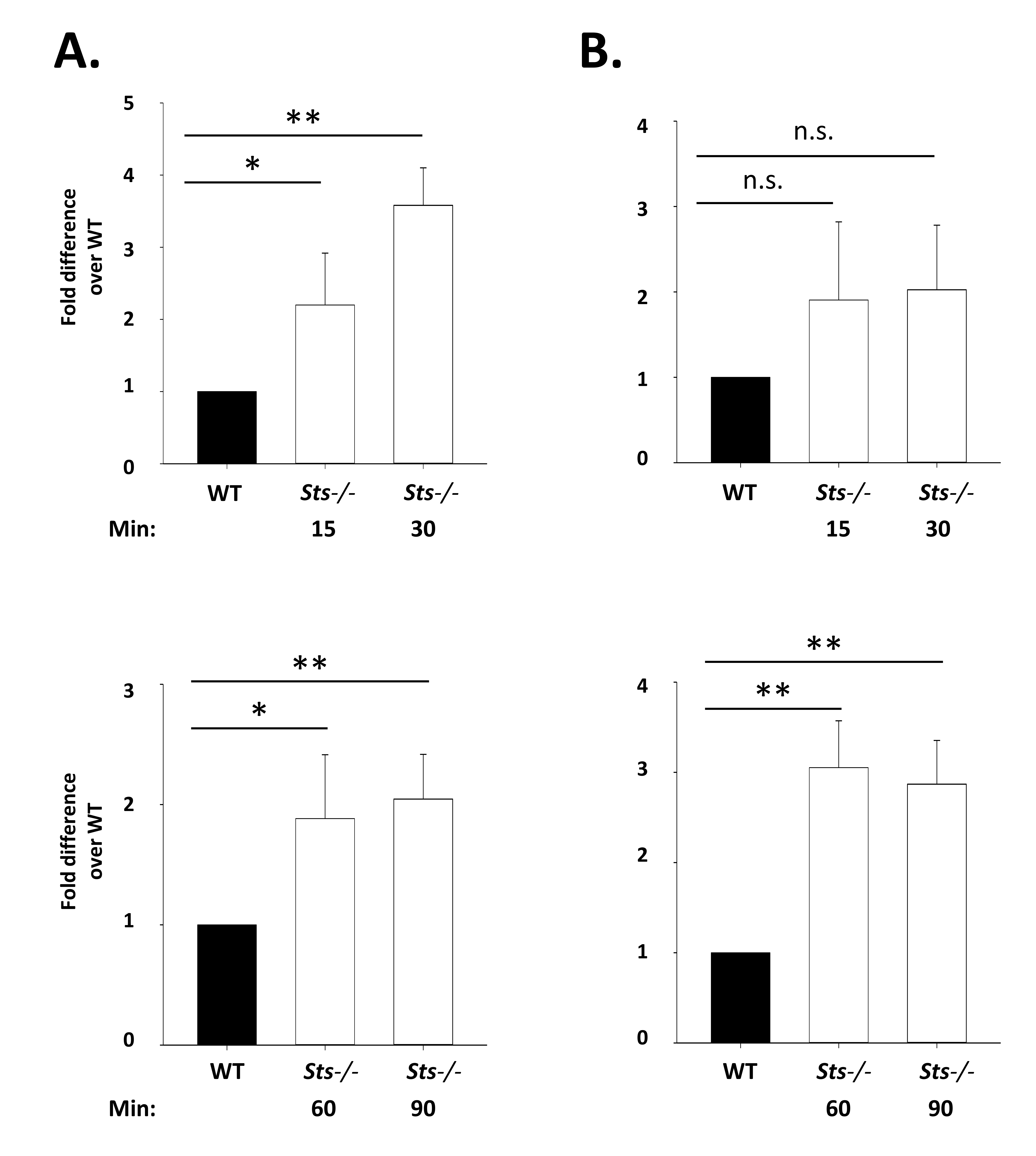

Supplement: FIG S5 [file mbo004183975sf5.tif]
